# SARS-CoV-2 viral proteins trigger pain via TLR2/4-MyD88 pathway

**DOI:** 10.3389/fnmol.2025.1163636

**Published:** 2025-06-16

**Authors:** Wenliang Su, Xinrui Wang, Minghui Gu, Qiwei Zheng, Jiawen Yu, Dongliang Mu

**Affiliations:** ^1^Department of Anesthesiology, Peking University First Hospital, Beijing, China; ^2^Department of Pharmacy, Beijing Chaoyang Hospital, Capital Medical University, Beijing, China; ^3^Department of Rehabilitation Medicine, Tongji Hospital, Tongji Medical College, Huazhong University of Science and Technology, Wuhan, Hubei, China; ^4^Shenzhen People's Hospital, Shenzhen, China; ^5^Department of Anesthesiology, Peking Union Medical College Hospital, Chinese Academy of Medical Sciences and Peking Union Medical College, Beijing, China

**Keywords:** SARS-CoV-2, TLR2, TLR4, MyD88, pain

## Abstract

Somatosensory disorders, especially pain, are prominent symptoms of COVID-19. Except for the viral infection process, SARS-CoV-2 viral proteins might be directly sensed by corresponding receptors, thereby triggering nociceptive signals in the dorsal root ganglion (DRG) and spinal dorsal horn (SDH). Behavioral assays were performed to screen out the nociceptive effects of the SARS-CoV-2 envelope protein (S2E) and spike protein receptor binding domain (S2S-RBD). Further investigation revealed that the genetic knockdown of TLR2 in the DRG and SDH significantly alleviated pain induced by both S2E and S2S-RBD. In contrast, the knockdown of TLR4 did not mitigate S2E-related pain but did reduce S2S-RBD-associated pain. Additionally, the knockdown of MyD88 effectively alleviated both mechanical and thermal pain induced by S2E and S2S-RBD. These findings indicate that the TLR2/4-MyD88 axis mediates SARS-CoV-2 protein-induced pain, and the interaction between viral proteins and neuro-immune receptors might serve as a key pathogenic factor in COVID-19 somatosensory disorders, suggesting a promising therapeutic strategy for these symptoms.

## Introduction

The pandemic Coronavirus disease 2019 (COVID-19), caused by severe acute respiratory syndrome coronavirus 2 (SARS-CoV-2), was initially identified as a respiratory disease characterized by symptoms of fever, cough, and fatigue. However, neurological symptoms, including paresthesia—such as anosmia, ageusia, and pain—have recently garnered significant attention (Su et al., 2020; Correia et al., 2020; He et al., 2021). Among these, pain typically manifests as headaches and musculoskeletal pain, though the underlying mechanisms remain unclear.

Studies indicate that a significant proportion of COVID-19 patients experience pain. For instance, Lin-Man Weng et al. reported that ~60% of COVID-19 patients experienced some form of pain, including headache, musculoskeletal pain, and chest pain (Weng et al., 2021). A systematic review and meta-analysis by Kamal et al. found that the prevalence of musculoskeletal pain among COVID-19 patients ranged from 14% to 33%, while headache was reported in 11%−34% of cases (Kamal et al., 2021). Headache is one of the most common pain symptoms. A study by Uygun et al. highlighted that headache was present in 13%−74% of COVID-19 patients. Additionally, chest pain associated with both respiratory and cardiac complications has been reported in a significant number of patients. Understanding the prevalence and types of pain experienced by COVID-19 patients can provide valuable context for probing the mechanism of SARS-CoV-2-induced pain and developing therapeutic strategies (Uygun et al., 2020).

Regarding the pain symptoms associated with COVID-19, it is essential to consider the dysfunction of the sensory system components induced by a direct viral infection and/or secondary neuroinflammation. SARS-CoV-2 has been detected in COVID-19 patients' cerebrospinal fluid and the brain with associated neuroinflammation (Luis et al., 2021; Li et al., 2021). SARS-CoV-2 is an enveloped, single-stranded RNA β-coronavirus composed of four major proteins: the spike (S2S), nucleocapsid (S2N), membrane (S2M), and the envelope (S2E) protein (Wang et al., 2020; Yang and Rao, 2021). Previous studies indicated that these viral proteins could be sensed by Toll-like receptors (TLRs), with S2E being sensed by TLR2 while S2S sensed by TLR2 and TLR4, subsequently triggering a storm of mediators (Frank et al., 2022; Zheng et al., 2021; Planès et al., 2022; Olajide et al., 2022; Segura-Villalobos et al., 2022; Tyrkalska et al., 2023; Khan et al., 2021).

Classically, MyD88 serves as the adaptor of TLRs signaling, controlling the downstream inflammatory cascade (Zheng et al., 2021; Planès et al., 2022; Khan et al., 2021; Su et al., 2021). Recent studies have shown that the myeloid differentiation factor-88 adaptor protein (MyD88) is also expressed in sensory neurons and spinal glia, which participate in pathological pain (Su et al., 2021; Liu et al., 2016). However, the specific role of MyD88 in COVID-19-associated pain remains unclear. In this study, we screened the nociceptive effects of three SARS-CoV-2 membrane proteins and distinguished the role of TLR2, TLR4, and downstream MyD88 in viral proteins induced pain.

## Methods

### Animals

C57BL/6 mice (2 months old, 20–30 g, provided by HFK Bioscience Co., Ltd, Beijing, China) were housed in a controlled environment (21 ± 4°C, standard 12-h light/dark cycle, 4–5 mice per cage). All animal experiments were conducted during light phase and approved by the Peking University Biomedical Ethics Committee experimental animal ethics branch. Both male and female mice were used in all studies. Animals were randomized to experimental groups, and the gender of used mice in each group was included in the figure legends.

### Behavioral assay

Mice were fully acclimated by being placed in the test chamber on the metal mesh 30 min before each behavioral assay. Behavioral tests were performed between 10 am and 12 am and all tested mice were housed in 12 h light/dark cycle. Mechanical allodynia was assessed by applying 0.4 g von Frey filaments to the plantar surface at a vertical angle for 3 s. Each mouse was tested 10 times and the percentage of paw withdrawal response was calculated.

Thermal hyperalgesia was evaluated by withdrawal latency in response to radiant heat (Hargreaves et al., 1988). Three repeat values were collected in each test with a 5 min interval, the mean of which was calculated as the final withdrawal latency. To avoid potential tissue damage, a cutoff time of 20 s was set for each thermal stimulus, with a 5 min interval between successive stimuli.

### Tail intravenous injection

The assay was performed as previously described (Turner et al., 2011). Mice were gently restrained using a restrainer, and the tail was warmed with a heat lamp to dilate the vein. Using a 1 mL syringe fitted with a 27-gauge needle, the solution was slowly injected into the lateral tail vein. Successful injection was confirmed by the absence of resistance and swelling at the injection site. Each injection was completed within 1–2 min. After wiping the tail with alcohol, a 100 μL volume of SARS-CoV-2 envelope protein (S2E, 5 μg, ENN-C5128, Acro Biosystems), SARS-CoV-2 membrane protein (S2M, 5 μg, 10-429, Prosci) or spike protein (S2S-RBD, 5 μg, 40591-V08H41, Sino Biological, Omicron B.1.1.529) solution buffered in PBS was administered intravenously.

### Small interfering RNA (siRNA) injection

TLR2 siRNA (siTLR2), TLR4 siRNA (siTLR4), MyD88 siRNA (si MyD88) or scramble RNA (sc RNA) (Keygen, Nanjing, China) was buffered in RNA enzyme free water. Intrathecal injection was performed using an insulin syringe to deliver reagents into the subarachnoid space between the L5 and L6 vertebrae. A rapid tail-flick response upon needle insertion was used to confirm proper needle placement. Behavioral assay was performed 3 days after si RNA injection.

### qRT-PCR

The lumbar dorsal root ganglion (DRG) and spinal dorsal horn (SDH) were collected and kept in the RNA later (Invitrogen, USA) overnight. Total RNAs were extracted using Trizol reagent (Invitrogen, USA), which were then reversely transcribed by RT Master Mix (Takara, Japan) according to the manufacturer's instructions. qRT-PCR amplifications were conducted by using CFX96™ Real-Time PCR Detection System (Bio-Rad, Hercules, California, USA) with SYBR premix Ex Taq™ (Takara, Japan). The housekeeping gene β-Actin was used for normalization. The primers were listed as follows: β-Actin, F-GTCCCTCACCCTCCCAAAAG, R-GCTGCCTCAACACCTCAACCC; MyD88, F-AGCAGAACCAGGAGTCCGAGAAG, R-GGGCAGTAGCAGATAAAGGCATCG; TLR2, F-TTTCTACTTTACCCAGCTCGCTCA, R-GGAACTGTCGGAGGTAGAGTTCG; TLR4, F-AAACTTGCCTTCAAAACCTGGC, R-ACCTGAACTCATCAATGGTCACATC.

### Statistical analysis

Data are presented as means with standard errors (mean ± SEM). Statistical analyses were performed using the SPSS software (version 17.0). A Student's *t*-test was used to evaluate the statistical significance of a difference between two groups. Comparisons for multiple groups or multiple time points were carried out using a two-way ANOVA with Bonferroni's *post hoc* test. *P* < 0.05 was considered as statistical significance in all analyses. The statistical analysis results are attached in Supplementary Table S1.

## Results

### The nociceptive effects of SARS-CoV-2 proteins

**Figure 1:**
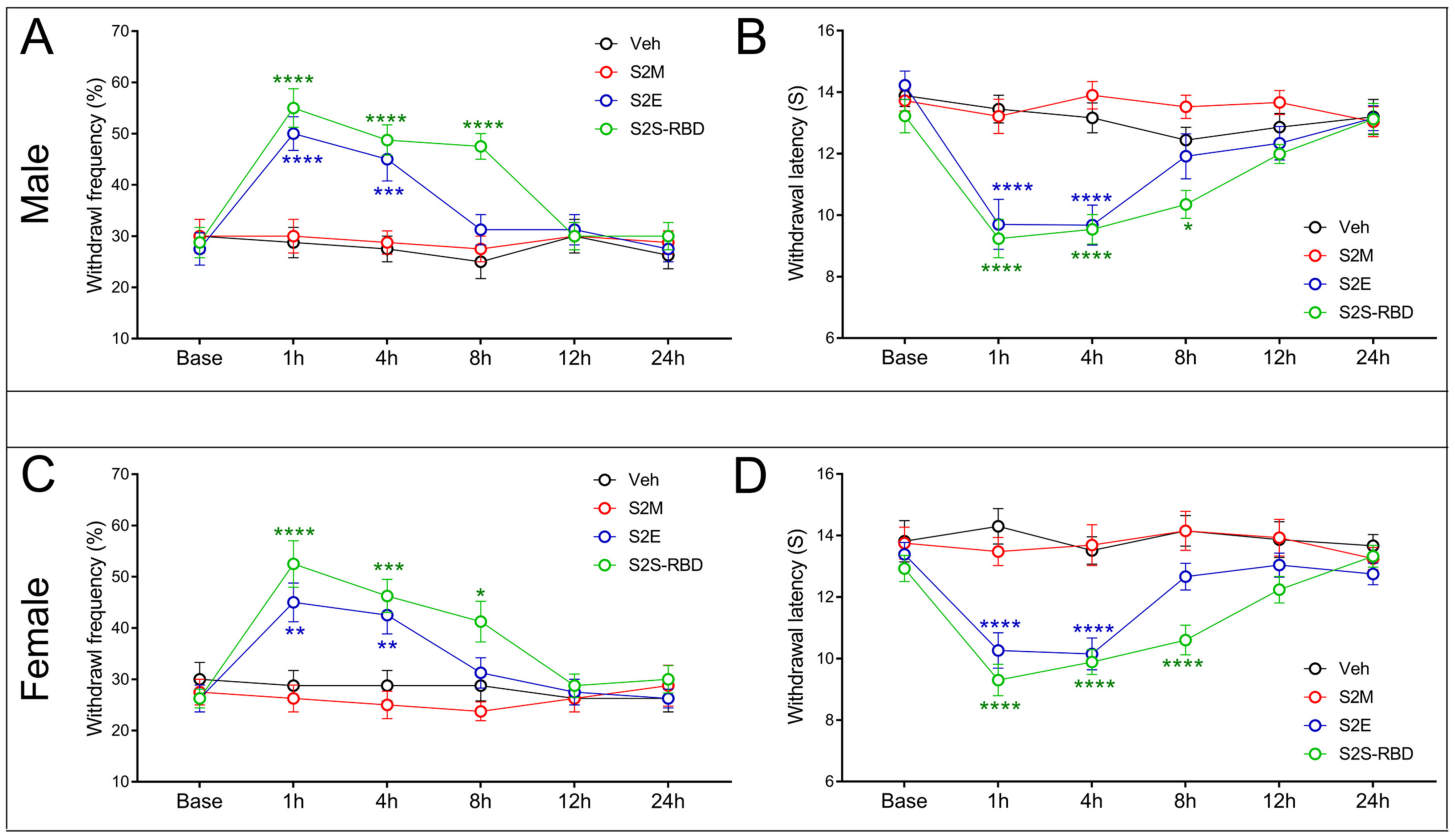
SARS-CoV-2 envelope protein (S2E) and SARS-CoV-2 spike protein receptor binding domain (S2S-RBD) induced mechanical and thermal pain. The time course of withdrawal frequency in 0.4 g von Frey test and withdrawal latency to thermal radiation for male mice **(A**, **B)** and female mice **(C**, **D)** receiving intravenous injection of SARS-CoV-2 membrane protein (S2M) (5 μg in 200 μL PBS), S2E (5 μg in 200 μL PBS) and S2S-RBD (5 μg in 200 μL PBS). *N* = 8 in each group, Two-Way ANOVA, Blue asterisk for S2E vs. Veh (PBS): ^**^P < 0.01, ^***^P < 0.001, ^****^P < 0.0001. Green asterisk for S2S-RBD vs. Veh (PBS): ^*^P<*0.05*, ^***^P < 0.001, ^****^P < 0.0001.

To figure out the nociceptive effects of SARS-CoV-2 proteins, we applied tail intravenous injection of S2M, S2E, and the spike protein receptor binding domain (S2S-RBD) in mice. Notably, S2M protein failed to induce mechanical allodynia or thermal hyperalgesia in either sex. Results showed that S2E could induce both mechanical allodynia and thermal pain within 4 h in male mice, with these effects extending to 8 h in the S2S-RBD treated group (Figures 1A, B). We observed that both S2E and S2S-RBD induced pain in male and female mice, and there were no statistically significant differences in the time course or intensity of pain behaviors between sexes (Supplementary Figure S1). These findings indicate that the pain-inducing effects of S2E and S2S-RBD are not sex-selective (Figures 1C, D).

### Distinct role of TLR2 and TLR4 in S2E and S2S-RBD induced pain

**Figure 2:**
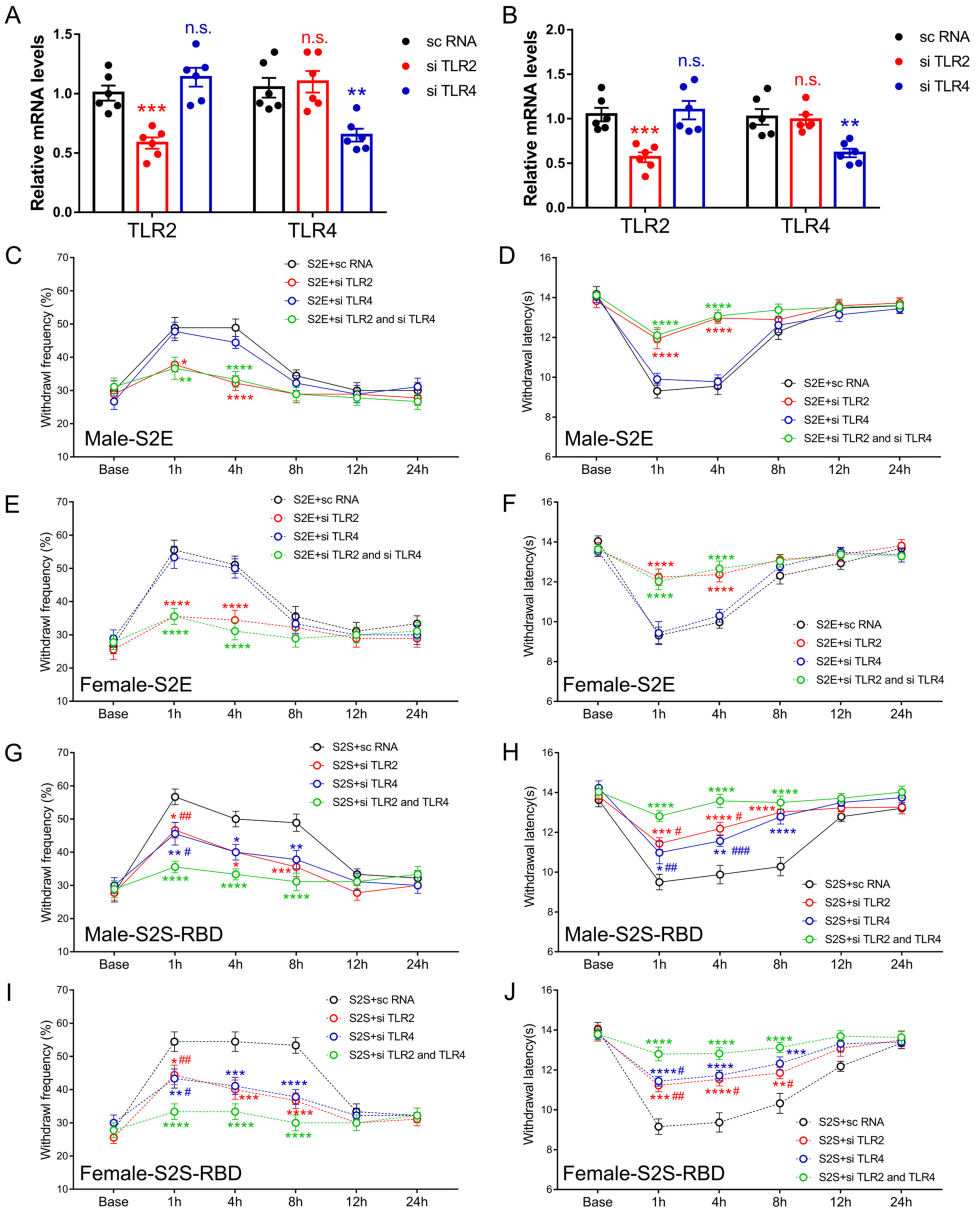
Distinct role of Toll-like receptor 2 (TLR2) and Toll-like receptor 4 (TLR4) in SARS-CoV-2 envelope protein (S2E) and SARS-CoV-2 spike protein receptor binding domain (S2S-RBD) induced pain. **(A, B)** mRNA expression of TLR2 and TLR4 in DRG **(A)** and SDH **(B)** for mice receiving intrathecal injection of si TLR2, si TLR4 and sc RNA. *N* = 6 in each group, One-way ANOVA, si TLR2 vs. sc RNA, ^***^P < 0.001 (Red asterisk); si TLR4 vs. sc RNA, ^**^P < 0.01 (Blue asterisk). **(C–F)** The time course of withdrawal frequency in 0.4 g von Frey test (C for male and E for female) and withdrawal latency (D for male and F for female) to thermal radiation for mice receiving intravenous injection of S2E (5 μg in 200 μL PBS) as pre-treated by si TLR2 and/or si TLR4. *N* = 8 in each group, Two-Way ANOVA: Red asterisk, si TLR2 vs. sc RNA; Green asterisk, si TLR2 and si TLR4 vs. sc RNA, ^*^P<*0.05*, ^**^P < 0.01, ^****^P < 0.0001. **(G–J)** The time course of withdrawal frequency in 0.4 g von Frey test (G for male and H for female) and withdrawal latency (H for male and J for female) to thermal radiation for mice receiving intravenous injection of S2S-RBD (5 μg in 200 μL PBS) as pre-treated by si TLR2 and/or si TLR4. *N* = 8 in each group, Two-Way ANOVA: Red asterisk, si TLR2 vs. sc RNA; Blue asterisk, si TLR4 vs. sc RNA; Red pound, si TLR2 vs. si TLR2 and si TLR4; Blue pound, si TLR4 vs. si TLR2 and si TLR4, ^*^P<*0.05*, ^**^P < 0.01, ^***^P < 0.001, ^****^P < 0.0001, ^#^*P*<*0.05*, ^##^*P* < 0.01, ^###^*P* < 0.001.

Given that SARS-CoV-2 viral proteins would be monitored by the innate immune system, we explored the role of classic pain-related innate immune receptors, specifically TLR2 and TLR4, in S2E and S2S-RBD-induced pain. The siTLR2 or siTLR4 was intrathecal injected (3 days before protein injection), combined or separately. The qPCR results confirmed that siTLR2 effectively downregulated TLR2 in both DRG and SDH, while siTLR4 reduced TLR4 expression (Figures 2A, B). The following behavior assay showed that siTLR2, but not siTLR4, could alleviate S2E-induced mechanical and thermal pain in both male and female mice. Moreover, the combined application of siTLR2 and siTLR4 did not mitigate S2E-related pain compared to siTLR2 alone (Figures 2C–F). As for S2S-RBD-induced pain, both siTLR2 and siTLR4 could effectively mitigate mechanical and thermal pain, with further alleviation observed when both were applied together (Figures 2G–J). Moreover, siRNA-mediated knockdown of TLR2 and TLR4 suppressed SARS-CoV-2 menbranal protein-induced pain to a similar extent in both male and female mice, with no significant sex differences. This suggests that the TLR2- and TLR4-mediated pain pathways are not sex-selective. These findings suggested that TLR2 could independently mediate S2E-induced pain, while S2S-RBD-induced pain involves the combined action of TLR2 and TLR4.

### MyD88 mediated pain induced by S2E and S2S-RBD

**Figure 3:**
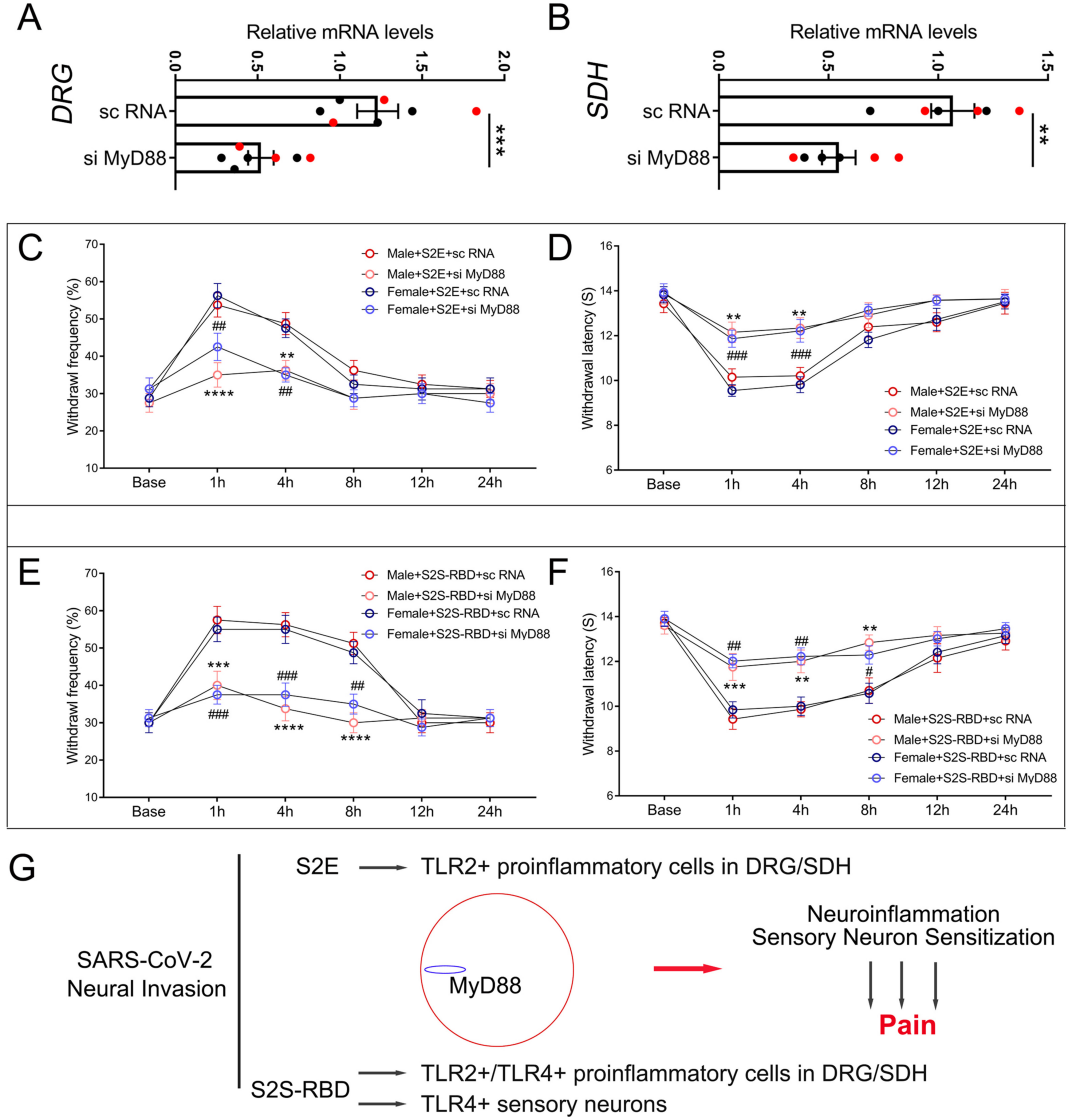
MyD88 mediated pain induced by SARS-CoV-2 envelope protein (S2E) and SARS-CoV-2 spike protein receptor binding domain (S2S-RBD). **(A, B)** mRNA expression of MyD88 in the DRG **(A)** and SDH **(B)** for mice receiving intrathecal injection of si MyD88 and sc RNA. Black circles represent data from male subjects, and red circles represent data from female subjects. The total number of subjects (N) ranges from 6 to 7. Student *t*-test, si MyD88 vs. sc RNA, **P < 0.01, ***P < 0.001. **(C, D)** The time course of withdrawal frequency in 0.4 g von Frey test **(C)** and withdrawal latency **(D)** to thermal radiation for male and female mice receiving intravenous injection of S2E (5 μg in 200 μL PBS) as pre-treated by si MyD88 or sc RNA. *N* = 8 in each group, Two-Way ANOVA: Male si MyD88 vs. sc RNA, **P < 0.01, ****P < 0.0001; Female si MyD88 vs. sc RNA, ^##^*P* < 0.01, ^###^*P* < 0.001. **(E, F)** The time course of withdrawal frequency in 0.4 g von Frey test **(E)** and withdrawal latency **(F)** to thermal radiation for male and female mice receiving intravenous injection of S2S-RBD (5 μg in 200 μL PBS) as pre-treated by si MyD88 or sc RNA. *N* = 8 in each group, Two-Way ANOVA: Male si MyD88 vs. sc RNA, **P < 0.01, ***P < 0.001, ****P < 0.0001; Female si MyD88 vs. sc RNA, ^#^*P*<*0.05*, ^##^*P* < 0.01, ^###^*P* < 0.001. **(G)** The graphical mechanism of MyD88 mediated SARS-CoV-2 viral proteins induced pain.

To determine the role of MyD88 in the pain symptoms induced by S2E and S2S-RBD, we administered MyD88 small interfering RNA (si MyD88) or scramble RNA (sc RNA) intrathecally, 3 days before protein injection. The qPCR results showed that si MyD88 significantly downregulated MyD88 expression in the DRG and SDH (Figures 3A, B). Further behavioral tests indicated that si MyD88 alleviated S2E-associated allodynia and thermal pain in male and female mice (Figures 3C, D). Meanwhile, si MyD88 injection also mitigated S2S-RBD-related pain compared to sc RNA (Figures 3E, F). Moreover, siRNA-mediated knockdown of MyD88 suppressed SARS-CoV-2 membranal protein-induced pain similarly in male and female mice, with no significant sex differences observed. This suggests that the MyD88-mediated pain pathway is not sex-selective. Collectively, these findings indicate that MyD88 mediated pain induced by SARS-CoV-2 viral proteins.

## Discussion

In this study, we first identified that SARS-CoV-2 membranal proteins, specifically S2E and S2S-RBD, could directly induce nociceptive effects. The inability of the S2M protein to induce inflammation, in contrast to the S2E and S2S proteins, may stem from its structural and functional properties. Unlike S2E and S2S proteins, which interact directly with host immune receptors such as TLR2 or TLR4, S2M primarily functions in viral assembly and budding, with limited exposure to the extracellular environment. It lacks classical pathogen-associated molecular patterns (PAMPs) that are recognized by pattern recognition receptors (PRRs), and does not exhibit viroporin activity or receptor-binding domains. Moreover, S2M protein has been reported to suppress type I interferon signaling, suggesting a potential immunomodulatory rather than proinflammatory role. Our findings, along with previous research, underscore the importance of the distribution of SARS-CoV-2 membranal proteins in understanding the pathophysiology of COVID-19. The SARS-CoV-2 membranal proteins could target multiple systems. In the respiratory system, the spike protein (S2S-RBD) is crucial for viral entry into host cells via the ACE2 receptor, predominantly expressed in lung epithelial cells (Li et al., 2020). In the central nervous system (CNS), evidence suggests that SARS-CoV-2 can penetrate the CNS, with viral RNA and proteins detected in cerebrospinal fluid and brain tissues of infected patients (Paniz-Mondolfi et al., 2020). This is associated with neurological symptoms such as anosmia, ageusia, and headache. The interaction of viral proteins with TLRs in the CNS may contribute to neuroinflammation and pain. In the cardiovascular system, the SARS-CoV-2 proteins have been detected in the heart and vascular tissues, potentially contributing to myocarditis, endothelial dysfunction, and thromboembolic events observed in COVID-19 patients (Tavazzi et al., 2020). And in other tissues, SARS-CoV-2 proteins are also detected in other tissues such as the gastrointestinal tract, kidney, liver, spleen, and even the skin (Cheung et al., 2020; Puelles et al., 2020). The broad distribution of viral proteins highlights the systemic nature of COVID-19 and the potential for widespread inflammatory responses.

Although S2E and S2S-RBD could directly induce nociceptive effects, the mechanisms underlying the pain induced by these proteins may differ, with MyD88 serving as a conserved signal in these processes. Briefly, the classic innate immune receptors TLR2 and TLR4 engaged in S2E and S2S-RBD-related pain, either separately or jointly. S2E could activate TLR2 signaling, mainly in the SDH microglia and DRG macrophage, and further contributed to the release of nociceptive mediators in DRG and SDH. Such as prostaglandins, bradykinin, histamine, and cytokines. MyD88 regulated the degree of TLR2 signaling activation and further proinflammatory cascade, with its inhibition alleviating S2E-associated pain. Apart from the above TLR2-related mechanism, S2S-RBD also interacts with TLR4^+^ cells, including sensory neurons, macrophages, microglia cells, and astrocytes, further triggering sensory sensitization and neuroinflammation (Frank et al., 2022; Zhao et al., 2021; Sahanic et al., 2023). Regardless of whether TLR2^+^ or TLR4^+^ cells were activated by viral S2E and S2S-RBD, MyD88 is a critical element in the SARS-CoV-2 protein-induced pain symptom (Figure 3G). These findings uncovered the central role of MyD88 in COVID-19 pain symptoms and suggested promising therapeutic strategies for managing pain associated with viral infection.

## Data Availability

The original contributions presented in the study are included in the article/Supplementary material, further inquiries can be directed to the corresponding author.

## Supplementary material

Supplementary Figure S1 SARS-CoV-2 envelope protein (S2E) and SARS-CoV-2 spike protein receptor binding domain (S2S-RBD) induced mechanical and thermal pain without sex differences. **(A, B)** The time course of withdrawal frequency in 0.4 g von Frey test and withdrawal latency to thermal radiation for male and female mice receiving intravenous injection of SARS-CoV-2 spike protein (S2S) (5 μg in 200 μL PBS). **(C, D)** The time course of withdrawal frequency in 0.4 g von Frey test and withdrawal latency to thermal radiation for male and female mice receiving intravenous injection of SARS-CoV-2 envelope protein (S2E) (5 μg in 200 μL PBS). **(E, F)** The time course of withdrawal frequency in 0.4 g von Frey test and withdrawal latency to thermal radiation for male and female mice receiving intravenous injection of SARS-CoV-2 membrane protein (S2M) (5 μg in 200 μL PBS). *N* = 8 in each group, Two-Way ANOVA, n.s., no significance, *P* > *0.05*.
