# Supplementary figures and images for: SARS-CoV-2 viral proteins trigger pain via TLR2/4-MyD88 pathway
Source: Front Mol Neurosci. 2025 Jun 16;18:1163636. doi: 10.3389/fnmol.2025.1163636 (PMC12206785; doi:10.3389/fnmol.2025.1163636)

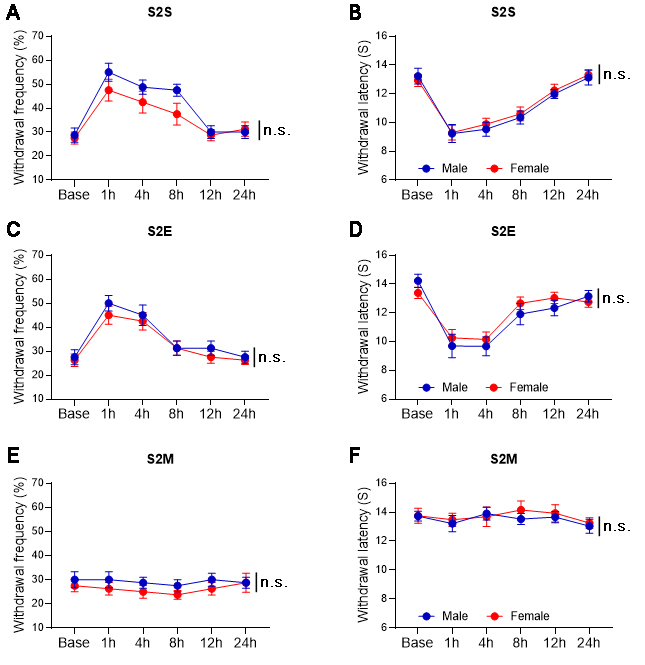

Supplement: Supplementary Figure S1 — SARS-CoV-2 envelope protein (S2E) and SARS-CoV-2 spike protein receptor binding domain (S2S-RBD) induced mechanical and thermal pain without sex differences. (A, B) The time course of withdrawal frequency in 0.4 g von Frey test and withdrawal latency to thermal radiation for male and female mice receiving intravenous injection of SARS-CoV-2 spike protein (S2S) (5 μg in 200 μL PBS). (C, D) The time course of withdrawal frequency in 0.4 g von Frey test and withdrawal latency to thermal radiation for male and female mice receiving intravenous injection of SARS-CoV-2 envelope protein (S2E) (5 μg in 200 μL PBS). (E, F) The time course of withdrawal frequency in 0.4 g von Frey test and withdrawal latency to thermal radiation for male and female mice receiving intravenous injection of SARS-CoV-2 membrane protein (S2M) (5 μg in 200 μL PBS). N = 8 in each group, Two-Way ANOVA, n.s., no significance, P > 0.05. [file Data_Sheet_1.zip › supplementary data/Figure S1.JPEG]
